# Supplementary material for: Coupling Effect of Non-Ignition Impact and Heat on the Decay of FOX-7
Source: Molecules. 2022 Nov 26;27(23):8255. doi: 10.3390/molecules27238255 (PMC9737319; doi:10.3390/molecules27238255)
Supplement: Supplementary file 1 [file molecules-27-08255-s001.zip › molecules-2026640-supplementary.pdf]

Coupling Effect of Non-Ignition Impact and Heat on the Decay of FOX-7

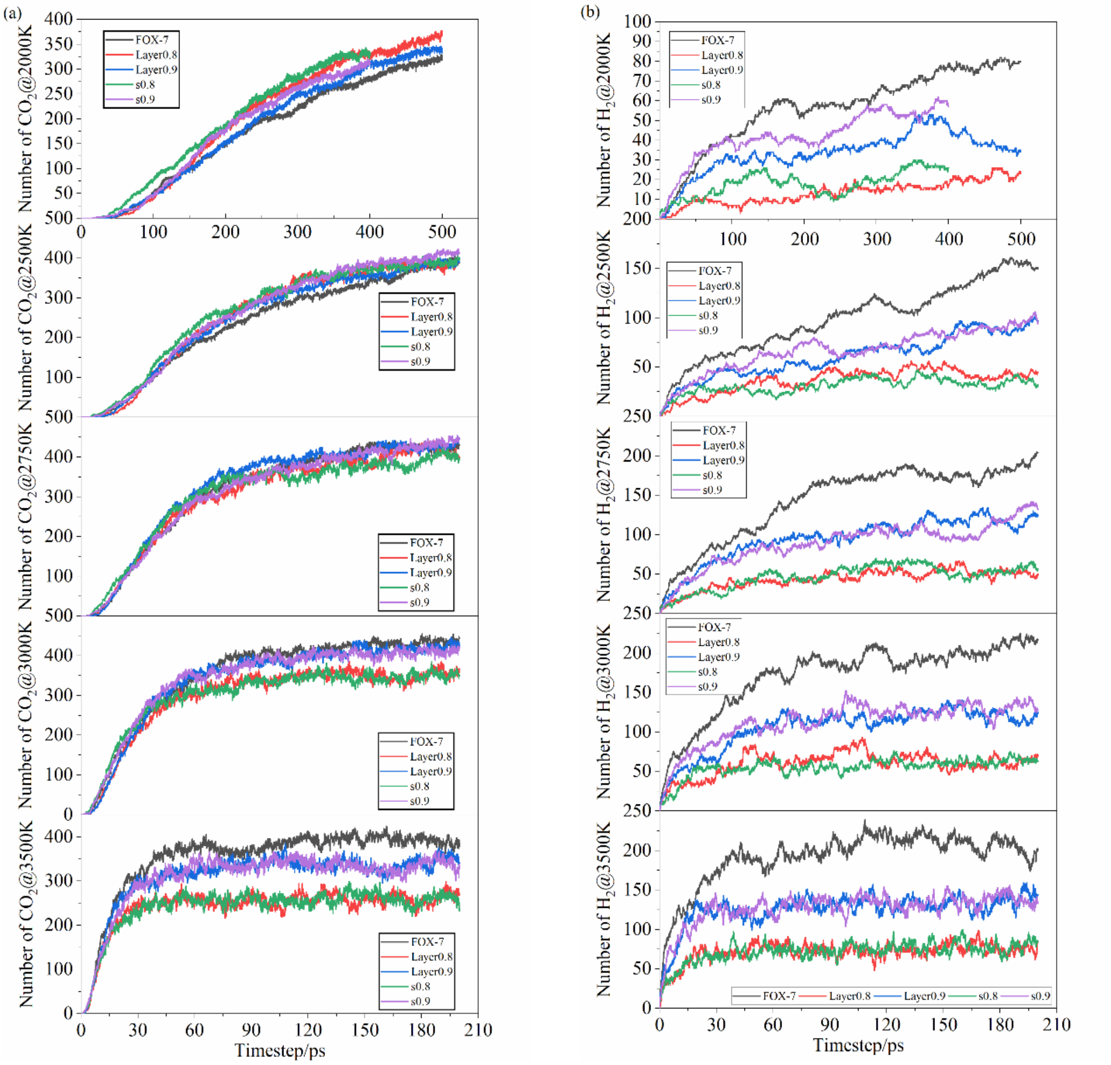

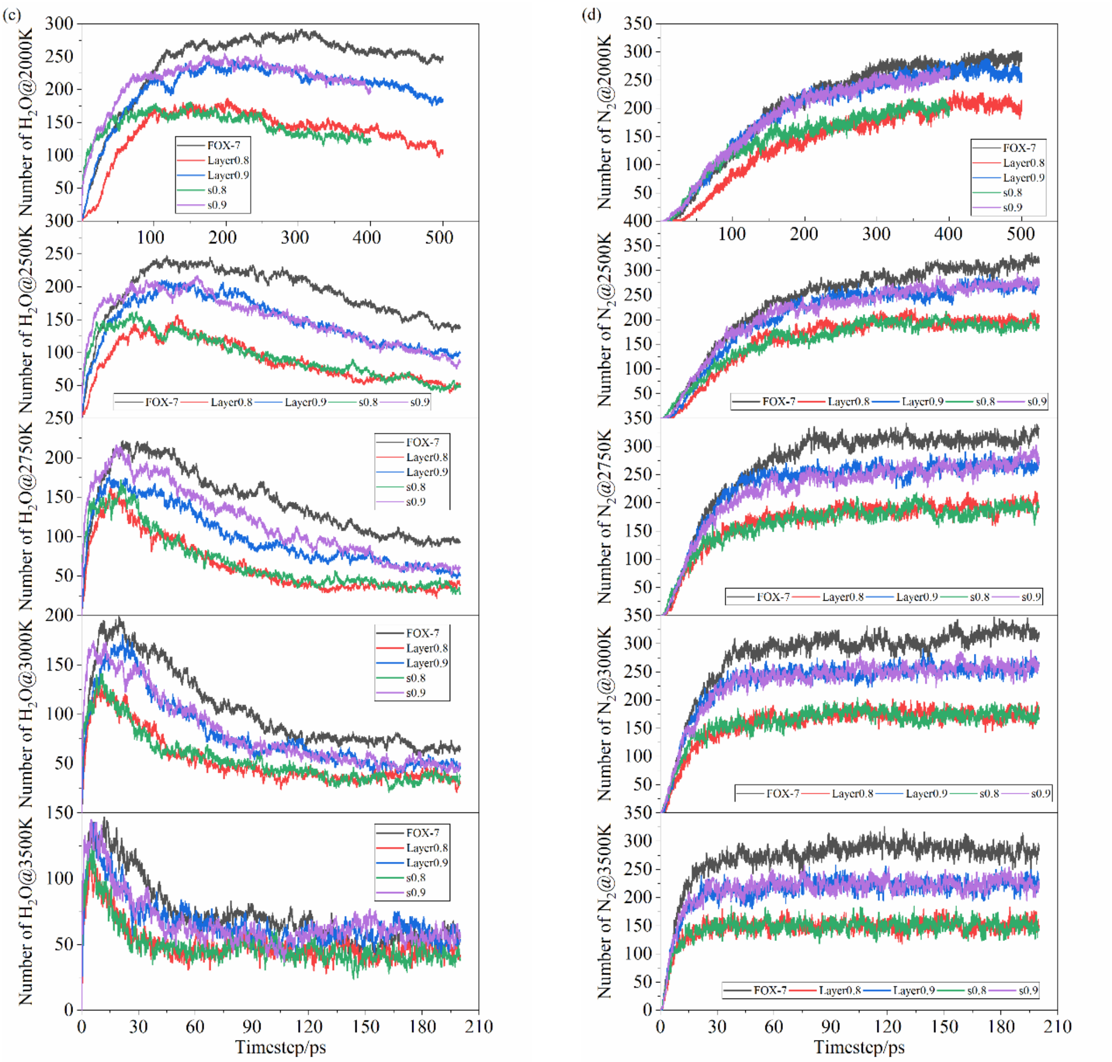
(c)
(d)
Number of H$_2$O@2000K
Number of H$_2$O@2500K
Number of H$_2$O@2750K
Number of H$_2$O@3000K
Number of H$_2$O@3500K
Number of N$_2$@2000K
Number of N$_2$@2500K
Number of N$_2$@2750K
Number of N$_2$@3000K
Number of N$_2$@3500K
FOX-7
Layer0.8
Layer0.9
s0.8
s0.9
Timestep/ps

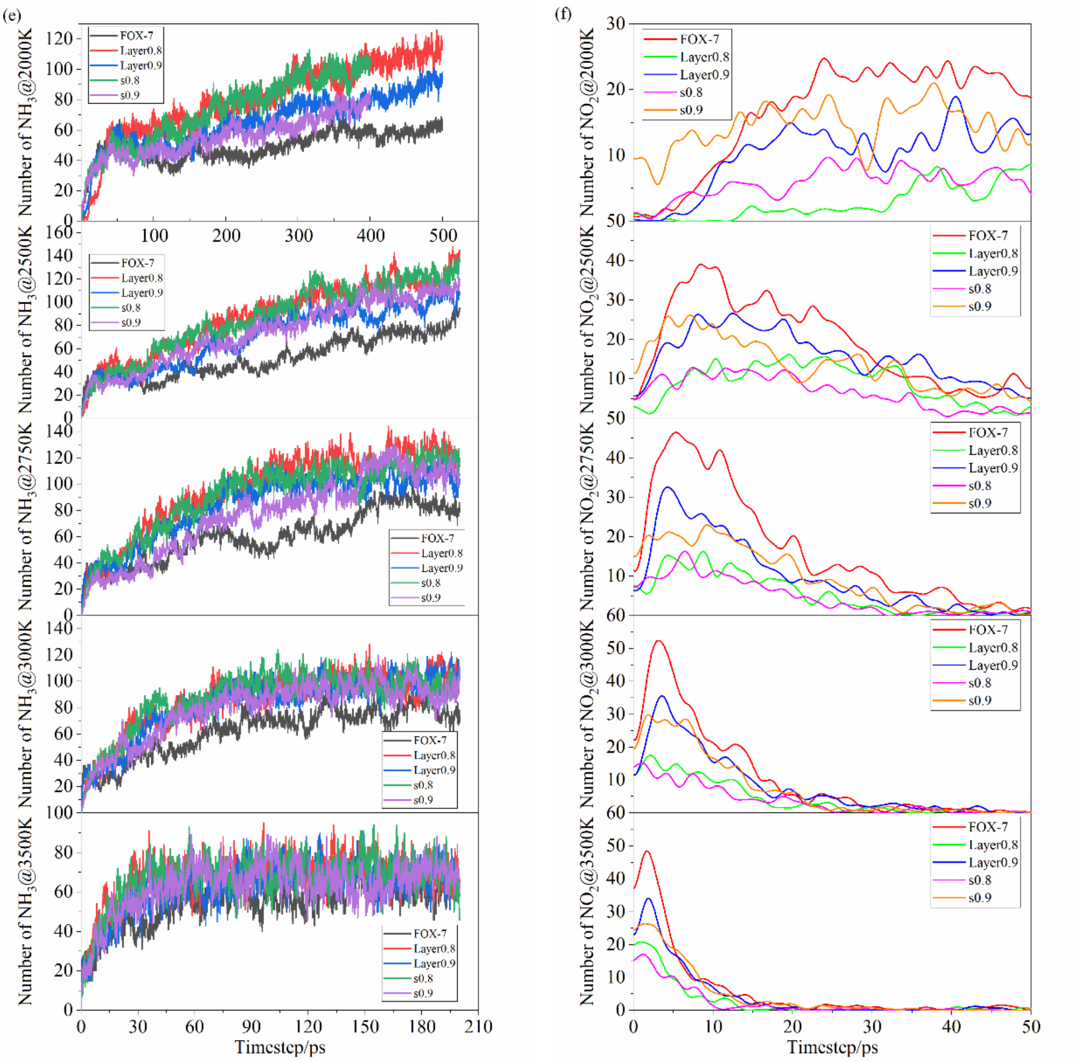

Figure S1 The evolution of the main related chemical species in all cases.

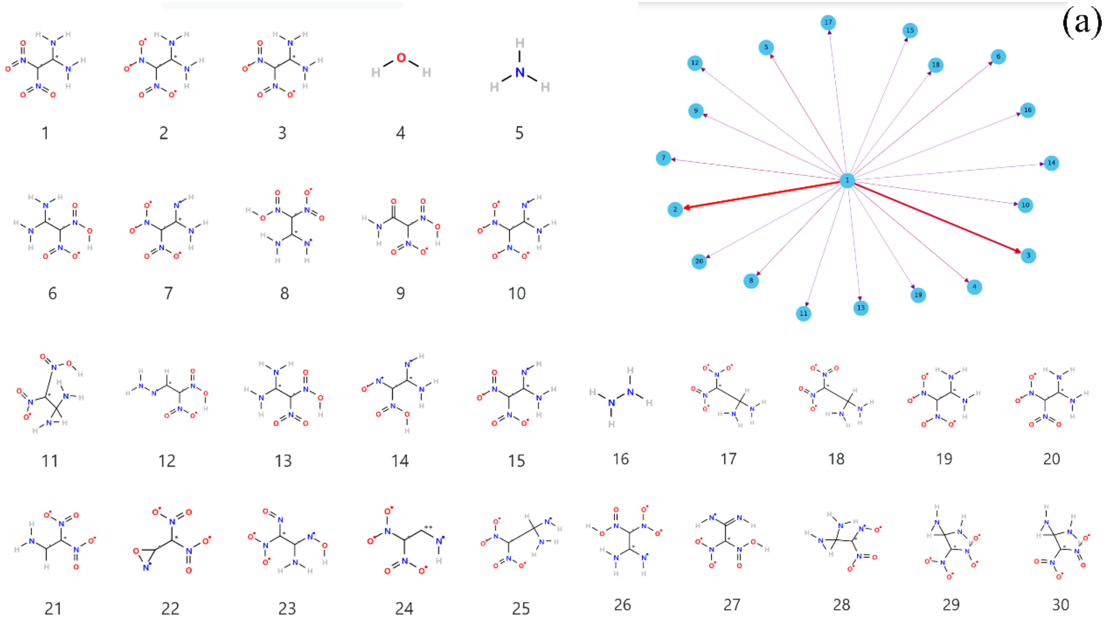
(a)

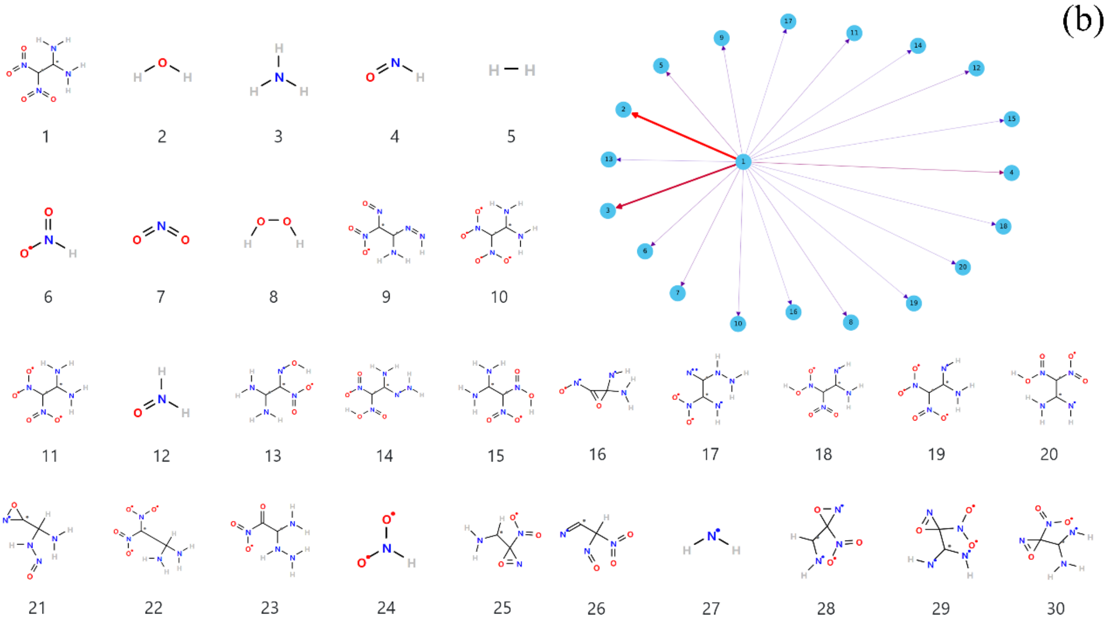
(b)

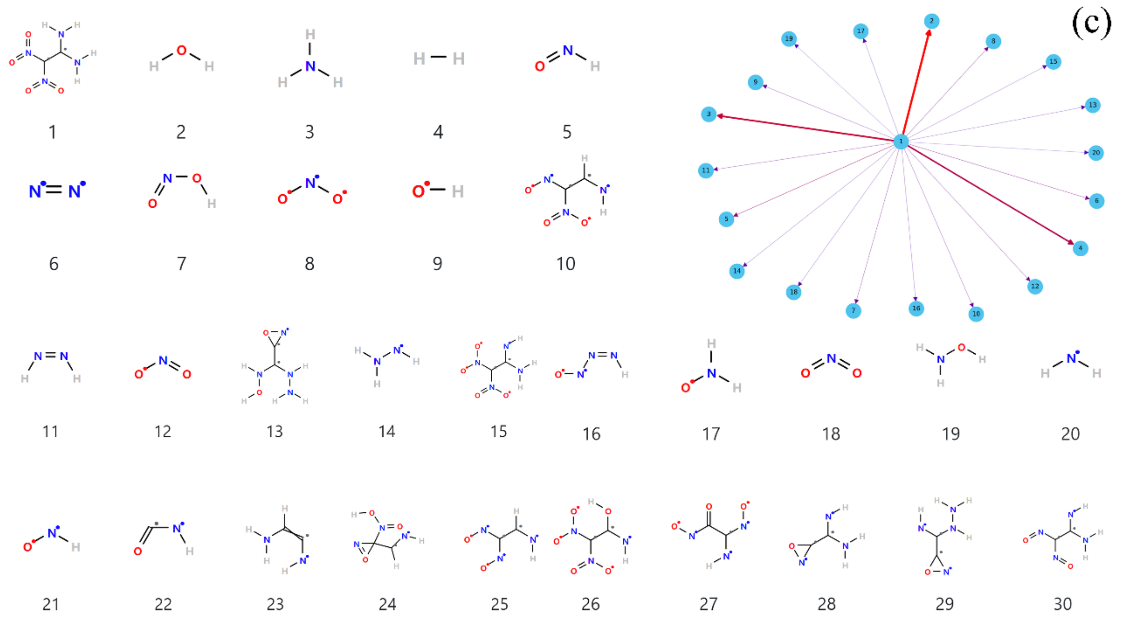
(c)
1 2 3 4 5
6 7 8 9 10
11 12 13 14 15 16 17 18 19 20
21 22 23 24 25 26 27 28 29 30

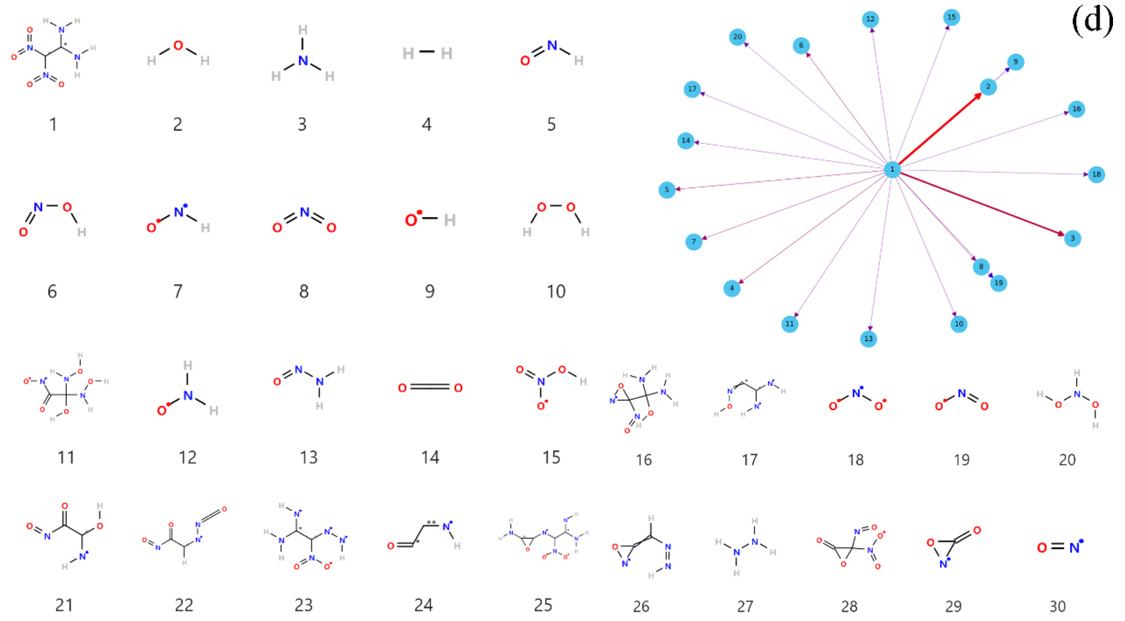
(d)
1 2 3 4 5
6 7 8 9 10
11 12 13 14 15 16 17 18 19 20
21 22 23 24 25 26 27 28 29 30

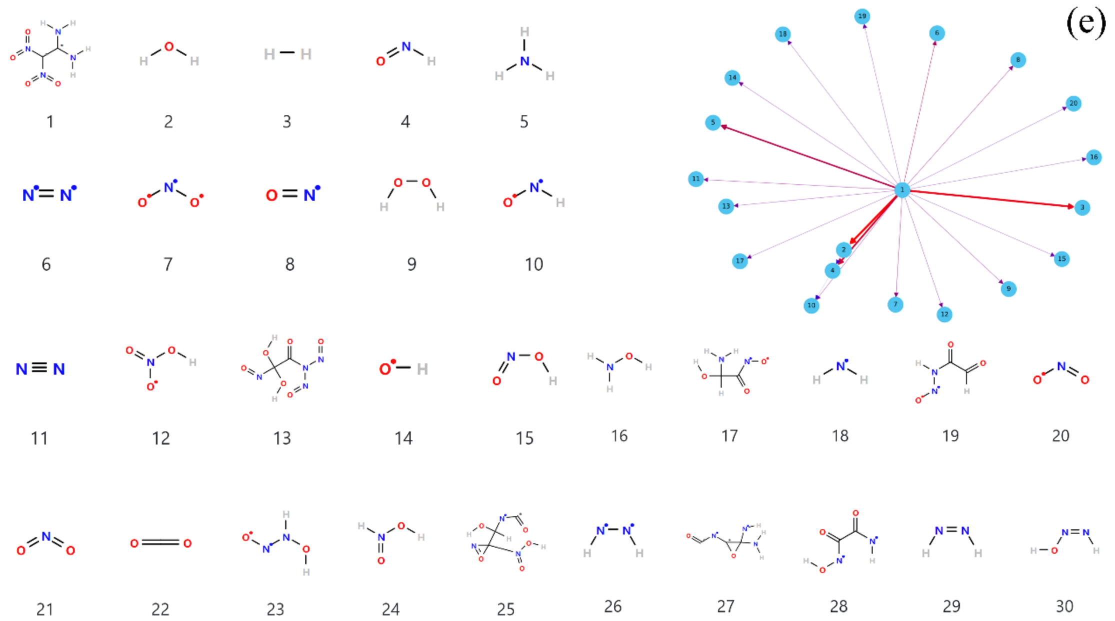

Figure S2. The initial reaction nets formed by the top 20 species under five temperatures.
